# CellCountCV—A Web-Application for Accurate Cell Counting and Automated Batch Processing of Microscopic Images Using Fully Convolutional Neural Networks

**DOI:** 10.3390/s20133653

**Published:** 2020-06-29

**Authors:** Denis Antonets, Nikolai Russkikh, Antoine Sanchez, Victoria Kovalenko, Elvira Bairamova, Dmitry Shtokalo, Sergey Medvedev, Suren Zakian

**Affiliations:** 1 A.P. Ershov Institute of Informatics Systems SB RAS, Novosibirsk 630090, Russia; russkikh@nprog.ru (N.R.); dmitry@nprog.ru (D.S.); 2 Novel Software Systems LLC, Novosibirsk 630090, Russia; 3 State Research Center of Virology and Biotechnology “Vector” Rospotrebnadzor, Koltsovo 630559, Russia; 4 Grenoble Institute of Technology ENSE3, 38031 Grenoble, France; antoine.sanches@gmail.com; 5 The Federal Research Center Institute of Cytology and Genetics SB RAS, Novosibirsk 630090, Russia; kov_r@ngs.ru (V.K.); bairamovaelvira@gmail.com (E.B.); medvedev@bionet.nsc.ru (S.M.); zakian@bionet.nsc.ru (S.Z.); 6 AcademGene LLC, Novosibirsk 630090, Russia; 7 St. Laurent Institute, Woburn, MA 01801, USA

**Keywords:** neural networks, fluorescent protein-based sensors, image analysis

## Abstract

In vitro cellular models are promising tools for studying normal and pathological conditions. One of their important applications is the development of genetically engineered biosensor systems to investigate, in real time, the processes occurring in living cells. At present, there are fluorescence, protein-based, sensory systems for detecting various substances in living cells (for example, hydrogen peroxide, ATP, Ca^2+^ etc.,) or for detecting processes such as endoplasmic reticulum stress. Such systems help to study the mechanisms underlying the pathogenic processes and diseases and to screen for potential therapeutic compounds. It is also necessary to develop new tools for the processing and analysis of obtained microimages. Here, we present our web-application CellCountCV for automation of microscopic cell images analysis, which is based on fully convolutional deep neural networks. This approach can efficiently deal with non-convex overlapping objects, that are virtually inseparable with conventional image processing methods. The cell counts predicted with CellCountCV were very close to expert estimates (the average error rate was < 4%). CellCountCV was used to analyze large series of microscopic images obtained in experimental studies and it was able to demonstrate endoplasmic reticulum stress development and to catch the dose-dependent effect of tunicamycin.

## 1. Introduction

A detailed study of the normal and pathological processes within cells is one of the key goals of modern biomedical research. Current methods of microscopic analysis and visualization of cellular structures, organoids, and molecules allow one to obtain large sets of images that require further processing and analysis. Thus, the development of effective and precise methods of qualitative and quantitative image processing is an urgent task necessary for the automation of microscopic image analysis and investigation of various impacts on the cells, including the screening of potential medicinal compounds [1]. These methods should effectively cope with the high variance of cell shapes and sizes, and with their tendency to form dense clusters, which makes cell counting and segmentation extremely difficult. Besides, such methods, especially those involving machine learning techniques, must be data-efficient because manual labeling of the datasets is a very tedious task [2,3].

The endoplasmic reticulum (ER) is a multifunctional cellular compartment responsible for protein synthesis, folding and processing. Protein folding in the ER may be impaired under different pathological conditions or stimuli. This condition is known as endoplasmic reticulum stress [4]. ER stress activates a complex signaling network, referred to as the unfolded protein response (UPR). It is known that ER stress is observed in many neurodegenerative diseases (such as amyotrophic lateral sclerosis, Parkinson’s disease, Alzheimer’s disease etc.) and has crucial functions with regard to immunity and inflammation [4,5]. In addition, UPR is also considered as a target in cancer therapy [6].

XBP1 protein is an important component of unfolded protein response. In ER stress, XBP1 mRNA undergoes specific splicing by the IRE1α protein. As a result, the active XBP1 protein is formed, which activates the genes necessary to compensate for ER stress [7]. Currently, these events can be detected with genetically engineered biosensors based on fluorescent proteins, changing their fluorescence intensity, spectral characteristics and/or localization, depending on molecular context. This approach allows real time visualization of the pathological processes occurring in living cells. Thus, specific sensory systems allowing one to visualize XBP1 activation in vitro and in vivo were developed [8,9]. However, the problem of reliable processing and statistical analysis of large sets of microscopic images to assess ER stress severity and to assess the efficiency of potential therapeutic compounds remained largely unsolved.

Here, we describe the development of a deep neural network for quantitative analysis of microscopic images of 293A cells, expressing the ER stress biosensor XBP1-TagRFP. The 293A cells are a subclone of the 293 human embryonic kidney (HEK) [10] line, which contains a stably integrated copy of the E1 gene that supplies the E1 proteins (E1a and E1b) required to generate recombinant adenovirus. This cell line is characterized by a high proliferation rate, ease of cultivation and ease of transformation by plasmid vectors. Cell line 293 and its subclones are widely used in biomedical research for studying the functions of genes, intermolecular interactions, signaling cascades, various intracellular processes, and testing of new genome editing tools [11,12,13,14,15]. Due to its properties, various subclones of the cell line 293 are used in screening studies, including the use of genetically encoded biosensors based on the fluorescent proteins [16,17]. The product of these studies is often data in the form of microscopic images, which are obtained using fluorescence microscopes or various high-performance imaging systems. The 293A cells have polymorphic non-convex shapes and variable sizes and they tend to form dense clusters, making it difficult to count and segment the cells. The classical image processing techniques (segmentation based on watershed algorithm, different edge-detection, and filtration procedures) were found to be insufficient for this task. Cell counting was realized using the approach based on the paper by Cohen et al. [18], aimed at a very similar research field. The authors proposed a way to count multiple small objects (as compared to the image size) related to the same category using fully convolutional neural networks (FCNN), which can predict the so-called redundant count maps. This approach can efficiently deal with non-convex overlapping objects, which are virtually inseparable when using conventional image processing methods. Using the redundant count maps approach also helps to decrease manual labeling work, since the weight sharing property makes fully convolutional networks significantly less hungry for data, and, moreover, with the selected approach, each object requires only a one-pixel mark. Cell counts predicted with our model were very close to expert estimates. We have also developed a web-application, CellCountCV (Cell Counting with Computer Vision), for counting the cells on phase-contrast microscopic images. CellCountCV was used to analyze large series of microscopic images obtained in experimental studies (3880 images from experiment #1 and 5208 images from experiment #2) and it was able to demonstrate endoplasmic reticulum stress development and to catch the dose-dependent effect of tunicamycin. Thus, this approach can be used for automation of both qualitative and quantitative data analysis in biosensor studies and their applications. The usage examples and the statistical analysis details can be found at: https://github.com/denatns/CellCountCV. 

## 2. Materials and Methods

### 2.1. Plasmids Construction and Production

The fragment of the XBP1 gene, which encodes the 26 bp intron sensitive to endoplasmic reticulum stress, was amplified with PCR using the following primers: XBP1-cDNA-F 5’-GCGCGGTGCGTAGTCTGGAGC-3’ and XBP1-cDNA-R 5’-GGTATATATGTGGTCAAAACG-3’ from human brain cDNA. The obtained PCR-product was cloned into the pGEM-T Easy vector (Promega). Several plasmid clones were Sanger sequenced and plasmid clones with 26 bp XBP1 intron were found. One clone with the spliced variant of the XBP1 gene was also found. The obtained plasmid clones (with unspliced and spliced XBP1 variants) were used as a matrix for PCR with primers XBP1-S-F5’ CAAGCTAGCGCCACCATGGACTACAAAGACGATGACGACAAGGAGAAAACTCATGGCCTTGTAGTTGAG-3’ and XBP1-S-R 5’-AATGGTACCCCCTAAGTCAATACCGCCAGAATCC-3’. XBP1-S-F primer contains the FLAG epitope sequence and Kozak consensus sequence. The obtained PCR product was cloned into the pcDNA 3.1/Hygro (-) (Invitrogen) vector using the restriction enzymes NheI and Acc65I. A TagRFP fragment without a start codon was obtained by PCR with TagRFP-woATG-F 5’-GTCGGTACCGTGTCTAAGGGCGAAGAGCTG-3’ and BFPX3-R5’ GCGCTTAAGTTAATTAAGCTTGTGCCCCA-3’ primers and pTagRFP-N vector (Evrogen) as a DNA source. TagRFP fragment was cloned into the pcDNA 3.1/Hygro (-)-XBP1 vector using the restriction enzymes Acc65I and AflII. In the results, two plasmid vectors were obtained: pCMV-XBP1-TagRFP, which contains the unspliced XBP1 fragment, and pCMV-XBP1-TagRFP_intdel, which contains the spliced XBP1 version and constitutively expresses TagRFP for use as a control. Transfection grade plasmid DNA was isolated using the PureLink HiPure Plasmid Miniprep Kit (Thermo Fisher Scientific).

### 2.2. 293A Cells Transfection

The 293A cells (Invitrogen) were transfected using polyethyleneimine (PEI, Santa Cruz Biotechnology). Cells were seeded at 60–70% confluency into a well of 6-well plate. In total, 2 µg of plasmid DNA and 14 µg of PEI were mixed into 280 µl of Dulbecco’s modified Eagle’s medium (DMEM) without FBS and antibiotics, incubated for 10 min, and added dropwise to the cells. The culture medium was changed to a fresh medium after 6 h of incubation.

### 2.3. 293A Cells Cultivation and ER Stress Induction

293A cells (Invitrogen) were cultivated in a medium consisting of DMEM/F12 (1:1), 10% fetal bovine serum, 200 mM GlutaMAX (Life Technologies), 1% penicillin/streptomycin solution (Life Technologies), and 1× non-essential amino acids (Lonza) at 37˚C and 5% CO_2_. In the first series of experiments, to induce endoplasmic reticulum stress, tunicamycin (Abcam) was added to the culture medium at a 10 μg/mL concentration and DMSO was added to the control wells at a 1% concentration. In the second series of experiments, tunicamycin was added to the different wells of a 6-well plate at 0, 5, 10 and 15 μg/mL concentrations and DMSO was added to the control wells at 0, 0.5, 1 and 1.5% concentrations. Tunicamycin and DMSO were added 24 h after transfection. The experiments for all concentrations were repeated twice.

### 2.4. Microscopic Images Acquisition

For producing the images, the Cell-IQ MLF imaging system (CM Technologies) in the Interinstitutional Shared Center of Cell Technologies SB RAS was used. The phase contrast mode was used to obtain cell images. For the detection of TagRFP, a Cy3-C (Ex/Em, 531 nm/593 nm) filter was used. In all cases, 10× objective was used. 

### 2.5. Dataset Preparation

We used 20 manually labeled images, split into training and validation sets with an 8:2 ratio. Each image was split into quadrants to reduce GPU memory usage. Simple augmentation, namely rotations of 90°, 180° and 270°, was performed to increase the available amount of training data. Finally, the model was tested on 24 images which were not seen by the model during the training and which were not used for hyperparameter tuning. The training and validation image sets were preprocessed by human experts who manually labeled the cells with one-pixel marks. 

### 2.6. Neural Network Model Training and Cell Counting

**Figure 1:**
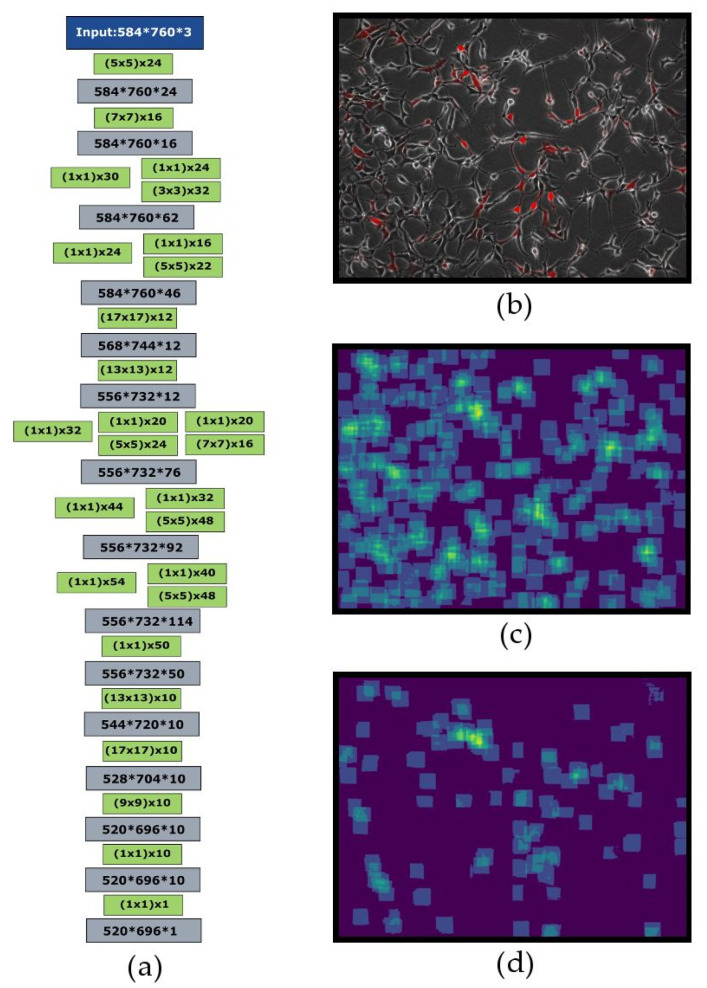
CellCountCV FCNN model architecture and microimage examples. The architecture of the developed FCNN model. Green blocks correspond to convolutional layers, where filter sizes are shown in brackets and additional numbers are the numbers of filters. (**a**) Numbers in grey blocks are output layer sizes. Batch-norm blocks are put after each convolutional or inception block and not shown; (**b**) Examples of original image section; (**c**) Corresponding count map; (**d**) The count map of the cells with red fluorescence above the selected threshold.

The model was greatly inspired by the Count-Ception network [18] and is also aiming to predict the redundant count maps. Each output pixel is supposed to count how many objects are present in its receptive field. Therefore, with this approach, any object is counted multiple times and that is why the result is called the “redundant count map”. The model consists of five inception blocks followed by additional convolutional layers. All activations are rectified linear units (ReLU). After each convolutional or inception layer, batch normalization was applied. Weights were initialized with the Glorot uniform approach. An overview of neural network architecture is presented in Figure 1. The model was trained with the Adam optimizer, with learning rate set to 0.001. Batch size was set to 1. It was trained for 400 epochs and then, the model with the lowest validation loss was evaluated on the test set. Fluorescent cell counting was implemented as a simple red channel binarization according to selected red signal intensity level (0–255; by default, the threshold was set to 100); the obtained mask was applied to the original grayscale image, then, the image was processed by the counting model, as usual.

### 2.7. Hardware and Software

The programs were implemented with Python programming language (v.3.6), using the following libraries: Keras (v.2.1.3) and Tensorflow (v.1.4.1) deep learning packages, which were used to create the FCNN model; computer vision and image processing library OpenCV (v.3.1.0) [19] and NumPy package (v.1.14.0) were used for image processing. Web service was created with Werkzeug (v.0.14.1) WSGI utility library and JSON-RPC Python library (v.1.10.3) and run in a docker container created using the Official Docker Image for TensorFlow (from https://hub.docker.com/r/tensorflow/tensorflow/) with the nvidia-docker plugin to enable GPU usage. API uses JSON-RPC protocol. It has a simple graphical user interface implemented with JavaScript library React (v.16.3.1). The model was trained on a workstation running under Ubuntu OS (v.16.04.5) with 32 Gb RAM, Intel® Core™ i7-7740 CPU and 2 NVIDIA GTX 1080 GPUs using an NVIDIA graphics driver (v.390.87) and CUDA v.9.1.85.

### 2.8. Availability

CellCountCV web-application can be accessed at http://cellcounter.nprog.ru. Web services can also be accessed programmatically through JSON-RPC calls to http://cellcounter.nprog.ru/api. CellCountCV usage examples, the pretrained FCNN model and the source code used for plotting and for statistical analysis can be found at https://github.com/denatns/CellCountCV.

## 3. Results

### 3.1. Stress Induction and Other Experimental Results

In this work, two series of experiments were carried out to induce the ER stress and to visualize the activation of the UPR system using a genetically encoded sensor XBP1-TagRFP.

### 3.2. FCNN Model for Cell Counting

The network architecture was greatly inspired by the original Count-Ception network described by Cohen et al. [18], which uses 1 × 1 convolutions. However, the cells and the images used in our study were different from those used to train Count-Ception. The original Count-Ception network was trained and evaluated on VGG, MBM, and Adipocyte cells, which have a more rounded shape than the 293A HEK cells used here. This increase in complexity made it harder for the original architecture to learn and caused us to develop a deeper FCNN architecture. The network structure is shown in Figure 1a. The two original steps consisting of chained inception layers are also separated by a chokepoint to decrease the size of subsequent hidden layers, and the network also ends with a second chokepoint which restores the size of the output to that of the input image. All images used in the current study were 8-bit TIFF files (in either RGB or grayscale), with a size of 1040 × 1392 px, which is at least 22 times the size of the images that had been used in the original paper by Cohen et al. [18]. As a fully convolutional neural network stacks the layers of all size levels of the image being processed, a vast amount of memory is required to work with big images. To counter this effect, each individual image was split into quarters and then, they were processed in parallel. The original images have a shape of 1040 × 1392 (×3 for RGB coding), their quarters have a shape of 520 × 696 (×3), and after padding, they are resized to 584 × 760 (×3). Each object is originally processed by the model with a 65×65 square kernel of ones. It is necessary to do image padding to be able to consider the cells located close to the image borders, thus, the images were extended by 32 zero pixels from each side. The FCNN, thus, produces the redundant count map and then, the number of the cells is inferred from the total sum of pixel values of the count-map and scaling it by the number of pixels in a chosen kernel (65 × 65 = 4225). This also allows us to reduce the total object counting error through averaging the individual object identification errors by the FCNN. An example of an original image and corresponding count map produced by the model is shown in Figure 1b.

The training and validation sets were at first labeled together, and then, split at an 8:2 ratio. Cell culturing and microscopic image acquisition are described in the corresponding Section 2. To provide more training data, we also used data augmentation: each original image was rotated by 90° three times and thus, three additional images were obtained from each of original ones. The training set series contained 16 images and 4 images were in the validation set. Final performance of our model was assessed with a separate testing set series containing 26 images; no part of which was seen by the model or used for hyperparameters’ tuning.

As there was very little difference between any pair of successive images, the final performance was evaluated using the images that were the most time-separated from each other. These labeled images were processed with our FCNN model. Then, the predicted cell counts were compared to counts provided by human experts.

### 3.3. Cell Counting Performance

To assess the performance of the produced FCNN model, we used the images from two series that were never seen by the model during the training. In total, 26 test images were randomly selected, and then, manually labeled by human experts. The total amount of cells in the test images exceeded 9000. The average error of predicted cell counts for testing images was about 3.12%, which is enough for the method to be considered reliable (Table 1). 

In 80% of testing images, the difference between the manual and predicted cell counts was less than 5%. The highest error percentage of 7.3% was obtained for an image with several clusters of cells, where even manual counting becomes error-prone. Finally, of all 9247 cells present in the testing dataset, the model counted 9037 of them, thus, the overall difference was 2.27%. This indicates that using a set of images (obtained e.g., from different fields of view) will result in higher precision due to averaging the cell counting errors between the neighbor images.

### 3.4. Web-Application CellCountCV for Cell Counting

The produced model was used to develop a simple web-application CellCountCV for counting the cells on microscopic images and to count the cells with fluorescence intensity above the user-selected threshold. Fluorescent cell counting was implemented using a simple binarization mask in the image’s red channel and its application as a filter to a grayscale image, then the image is processed with the usual counting model. Web-service API was implemented with JSON-RPC protocol and we also created simple graphical user interface using React JavaScript library. It accepts a microscopic image and red intensity threshold and returns the cell count and the number of cells with red fluorescence. It was hosted on our institution’s (IIS SB RAS) server and can be accessed at http://cellcounter.nprog.ru. Besides, it can be used directly through JSON-RPC calls to http://cellcounter.nprog.ru/api. In such a case, the image should be encoded as a Base64 string.

### 3.5. Automation of Stress Induction Analysis in 293A Cells with CellCountCV

Images from two ER stress induction time course experiments were processed with CellContCV and the obtained cell counts were analyzed. The processing of a single image file on average required about 8.26 s on a laptop with NVIDIA GTX 960M GPU and 3.46 s on a computing station with NVIDIA GTX 1080 Ti GPUs. 

### 3.6. Endoplasmic Reticulum Stress Induction Analysis with CellCountCV 

**Figure 2:**
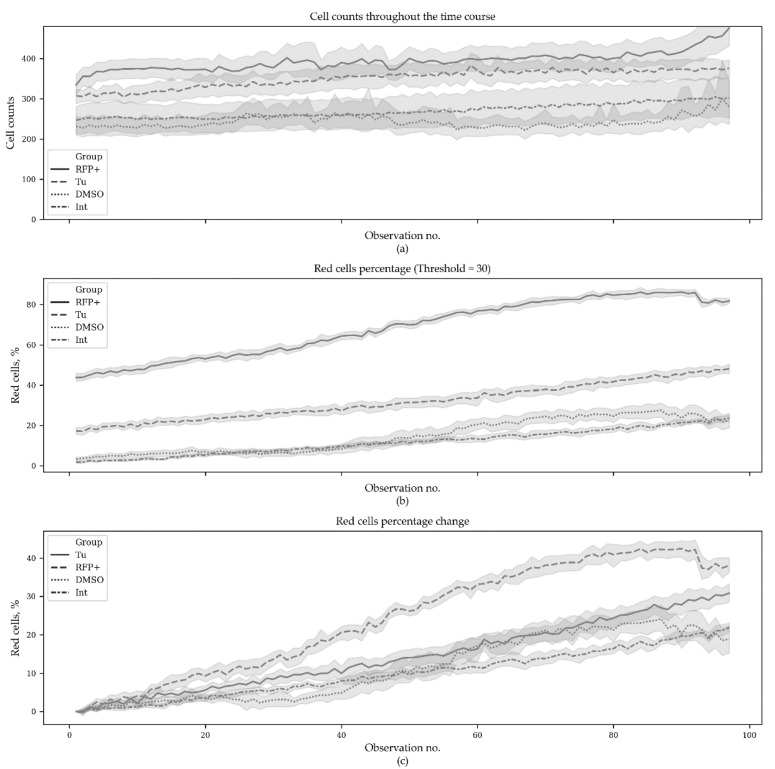
Cell counts and percentage of the red cells during the ER stress time course. In total, 3880 microscopic images were analyzed (970 images per group with 10 fields of view per time point). Tu—experimental group, where ER stress was induced with tunicamycin (10 μg/ml); TagRFP+—positive control group with constantly expressed TagRFP; DMSO—negative control group of transfected cells with DMSO added (tunicamycin solvent); Int (intact)—negative control group of transfected cells. (**a**) CellCountCV was used to estimate the cell counts; (**b**) red cells were counted at red intensity threshold set to 30 (out of 255) and the percentage of red cells was calculated; (**c**) the initial red cells percentage was subtracted from all subsequent values for each field of view. The horizontal axes correspond to observation number.

The first series contained 3880 microscopic images (970 images per group with 10 fields of view per time point). This series contained the following groups: Tu—experimental group where ER stress was induced with tunicamycin (10 μg/mL); TagRFP+—positive control group with constantly expressed TagRFP; DMSO—negative control group of transfected cells with DMSO added (tunicamycin solvent); Int (intact)—negative control group of transfected cells. The obtained results—cell counts, red cells percentage and red cells percentage change per group per timepoint—are presented in Figure 2. 

**Figure 3:**
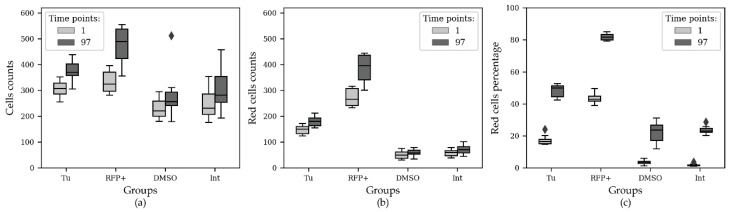
Cell counts (**a**), red cell counts (**b**) and red cells percentage (**c**) in the beginning and at the end of ER stress time course. Tu—experimental group where ER stress was induced with tunicamycin (10 μg/mL); TagRFP+—positive control group with constantly expressed TagRFP; DMSO—negative control group of transfected cells with DMSO added (tunicamycin solvent); Int (intact)—negative control group of transfected cells (N = 10).

The original data can be found in Table S1. Figure 3 demonstrates the same results but only for the starting and the ending time points.

Statistical analysis was performed with the non-parametric Mann–Whitney test and Bonferroni *p*-value adjustment for multiple comparisons. Since the initial percentages of red cells were significantly different between the groups (excepting the negative controls), the initial values were subtracted from the subsequent ones. It was found that at the end of experiment, the red cell percentages were significantly different between the groups (*p* < 0.005), except in the negative controls (*p* = 0.8191) (Table 2). 

Next, we performed regression analysis using the Generalized Estimating Equation (GEE) approach. GEE is a general statistical approach to fit a marginal model for longitudinal/clustered data analysis [20]. We used Poisson distribution to model count outcomes—red cells count per 1000 cells—with autoregressive covariance structure. GEE models were built with the statsmodels package (v. 0.10.1) [21] for Python. The model demonstrated statistically significant distinctions in all but the negative control groups (Int and DMSO) and also demonstrated significant changes in red cells counts with time (Table 3). The source code used to produce the plots and to perform the analysis can be found at https://github.com/denatns/CellCountCV.

### 3.7. Endoplasmic Reticulum Stress Induction and Tunicamycin and DMSO Dose-Effect Analysis with CellCountCV 

**Figure 4:**
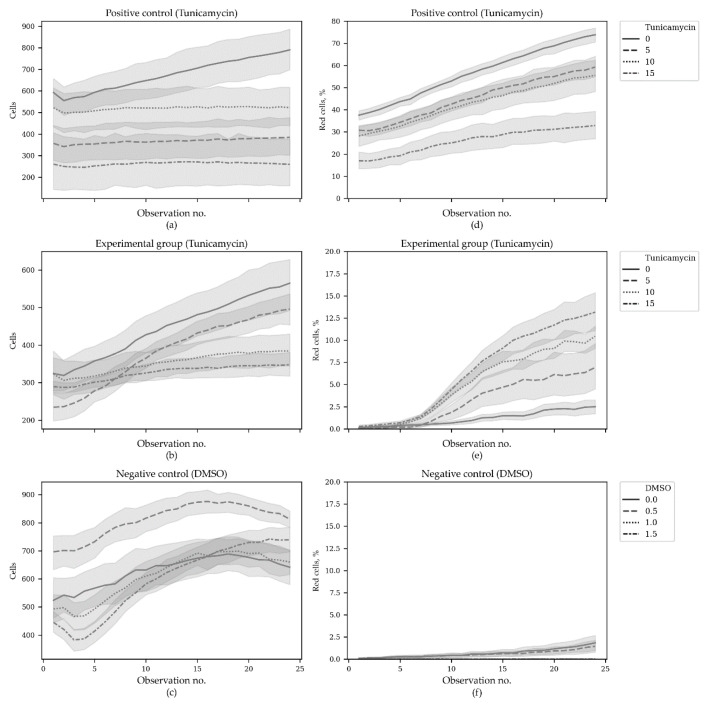
Cell counts and percentage of the red cells during the second ER stress time course experiment. In total, 5208 microscopic images were analyzed. Positive control—a group of cells that constantly expressed TagRFP; experimental group—the cells with TagRFP expression induced under ER stress conditions. These two groups were treated with different tunicamycin concentrations (µg/mL). Negative control group—the ER-responsive cells treated with different DMSO concentrations (%). Panels (**a**–**c**) show the cell counts throughout the time course and panels (**d**–**f**)—the red cells percentage. N = 18. The horizontal axes correspond to observation number.

In total, 5208 microscopic images were analyzed. A group of cells, constantly expressing TagRFP, was used as a positive control. The cells with TagRFP expression induced under ER stress conditions are referred to as the experimental group. These two groups were treated with different tunicamycin concentrations. The negative control group were the ER stress-responsive cells treated with different DMSO concentrations (tunicamycin solvent). Cells were counted and the number of red cells was obtained with CellCountCV; the percentages of red cells were calculated for each group in 20 fields of view at 24 time points (Figure 4). 

**Figure 5:**
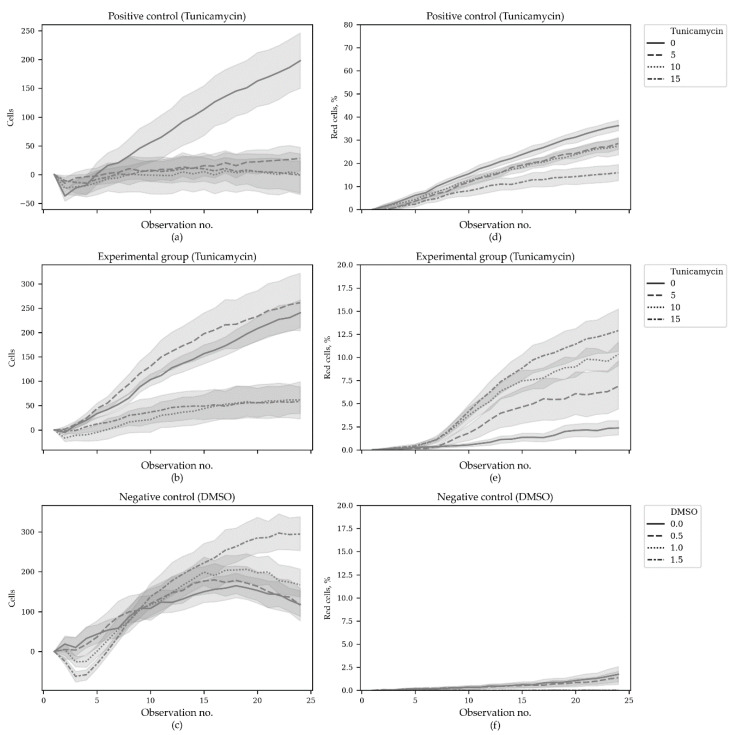
Cell counts and red cells percentage changes during the second ER stress time course experiment. In total, 5208 microscopic images were analyzed. Positive control—a group of cells that constantly expressed TagRFP; experimental group—the cells with TagRFP expression induced under ER stress conditions. These two groups were treated with different tunicamycin concentrations (µg/mL). Negative control group—the ER-responsive cells treated with different DMSO concentrations (%). Panels (**a**–**c**) show the cell count change throughout the time course and panels (**d**–**f**)—the change of red cells percentage. N = 18. The horizontal axes correspond to observation number.

Since the initial cell counts and percentages of red cells were significantly different between (and even within) the groups, the initial values were subtracted from the subsequent ones, obtained for the corresponding fields of view. The changes in total cell counts and red cell percentages throughout the time course are shown in Figure 5.

Statistical analysis was also performed using a non-parametric Mann–Whitney test with Bonferroni *p*-value adjustment. The results of the statistical comparison demonstrated significant distinctions between the groups. There were statistically significant distinctions between different tunicamycin and DMSO doses (Table 4). 

**Figure 6:**
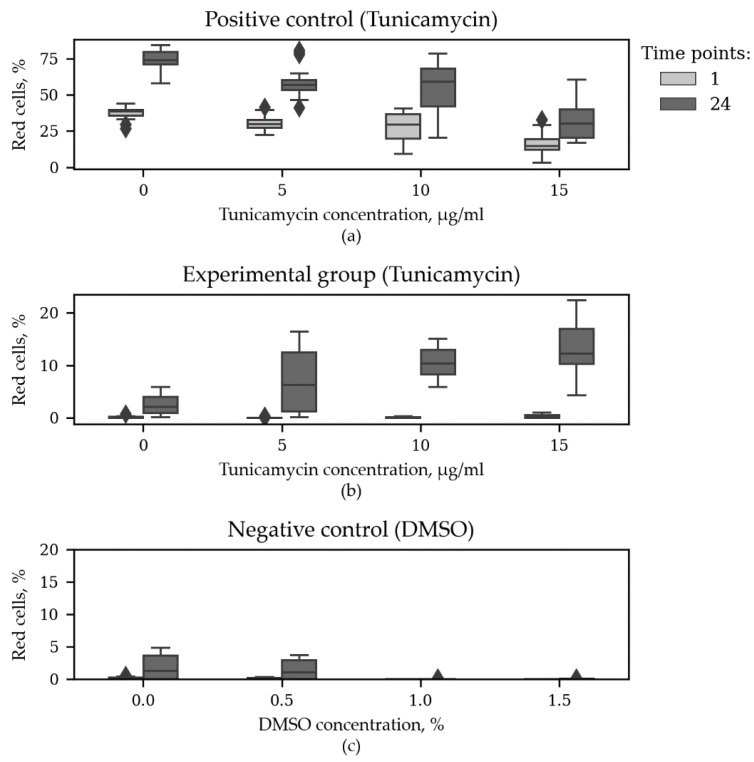
Red cell percentage in the beginning and at the end of the second ER stress time course. (**a**) Positive control group with constantly expressed TagRFP treated with different concentrations of tunicamycin; (**b**) Experimental group responsive to ER stress conditions treated with different concentrations of tunicamycin; (**c**) Negative control group treated with different concentrations of DMSO. N = 18. The horizontal axes correspond to Tunycamycin (**a**,**b**) and DMSO (**c**) concentrations.

It is of interest that in the positive control group—in cells constantly producing TagRFP—the increased tunicamycin concentration decreased the percentage of red cells, while in the experimental group, the tunicamycin dosage had the opposite effect (Figure 6).

To investigate the dose-effect of tunicamycin, we performed regression analysis using the generalized estimating equation (GEE) approach. Here, we also used Poisson distribution to model the count outcome—red cell count per 1000 cells—with autoregressive covariance structure. In total, 3480 observations were used, grouped into 145 clusters (according to group, tunicamycin dose and a field of view; cluster sizes were equal to 24 time points). DMSO-treated negative controls were excluded from the GEE analysis. The model was built with the statsmodels package for Python. The following factors were considered: Tu—tunicamycin concentration, Intron—intron present in the experimental group of cells and lacking in the positive controls, Time—a factor of observation time. The model demonstrated that all the factors had significant effect and that interactions of Tu and Intron, and Intron and Time were also significant (Table 5). 

Thus, the regression models also support our conclusions from Mann–Whitney tests about opposite effects of tunicamycin dosage in the experimental group and in the positive control. The original data can be found in Table S2. The source code used to produce the plots and to perform the analysis can be found at https://github.com/denatns/CellCountCV.

## 4. Discussion

With genetic engineering techniques, cellular models can be turned into biosensors to visualize cellular stress induced by particular treatment options. Currently, application of cellular models for studying the mechanisms of different pathogenic conditions, human diseases, and for developing new efficient therapeutics is actively evolving. There is special interest in cellular models that use the reporter systems based on the expression of fluorescent proteins. In such experiments, the presence of a fluorescent signal, a change in the spectral characteristics and/or intensity of fluorescence, or a change of signal localization within the cell can be detected. To increase the reliability of obtained data, it is necessary to process a large number of microimages. Thus, the researchers have to use either costly proprietary cell counting systems or semi-automated or manually operated software packages. This leads to significant time costs and generates the influence of the human factor on the result of the study. We also tried to process these images with either ImageJ [22] or with classical computer vision techniques such as watershed algorithm etc., realized in OpenCV library, but the obtained solutions were found to be inaccurate. That is why we decided to make a dedicated neural network-based model for processing microimages. To develop a prototype of software for automation of endoplasmic reticulum stress assessment, we used 293A HEK cells transfected with a plasmid expressing the biosensor XBP1-TagRFP and treated with various concentrations of endoplasmic reticulum stress inducer tunicamycin. 

The developed FCNN model was integrated into a simple web service named CellCountCV. It was found to effectively deal with high variance of cell shapes and sizes and the tendency to form dense clusters. It was also found to be data-efficient as the redundant count maps approach is less hungry for data, and each object requires only a one-pixel mark, making data-labeling less tedious and time consuming. Cell counts predicted with our model were very close to expert estimates. Our model can be used for automation of processing of large sets of microimages. The processing of a single image file on average required about 8.26 s on a laptop with NVIDIA GTX 960M GPU, and about 3.46 s on a computing station with NVIDIA GTX 1080 Ti GPUs. Thus, the whole set of 5208 images was processed in several hours. If image processing with a counting model runs in parallel with image acquisition, then all the results will be ready at the end of the experiment without any significant time delays. The CellCountCV application can be used either through the simple web user interface at http://cellcounter.nprog.ru or programmatically through JSON-RPC calls to http://cellcounter.nprog.ru/api. Some additional examples and the source code used for plotting and to perform the analysis can be found at https://github.com/denatns/CellCountCV. 

The FCNN approach used here also makes it possible to adapt the models for counting any homogenous small objects using the user labeled data. In the near future, we plan to extend CellCountCV functionality with additional color filters for fluorescence detection and to create a module for model adaptation to allow researchers to count objects of interest using their images. 

Obviously, further development requires the combination of both biosensors and a computational model trained on relevant cellular models, for example, on differentiated derivatives of patient-specific induced pluripotent stem cells [23]. Induced pluripotent stem cells (iPSC) are widely used in biomedical research and they are especially useful for developing cellular models of human diseases. Such models, based on iPSC, allow one to study the molecular mechanisms of pathogenesis, to search for target molecules, and to screen potential medicinal compounds against human diseases that currently have no efficient therapeutic medications. Such models can also be used to develop personalized and patient-oriented treatment strategies. Here, the 293A HEK cell line was used, which is not relevant to any particular disease. However, this model can be used for primary screening of substances, as was shown with the UPR pathway example.

## 5. Conclusions

The study of biological processes and the search for ways to influence them requires the creation of highly effective tools for automatic processing of microscopic images. We have developed a web-application, CellCountCV (Cell Counting with Computer Vision), for counting the cells on complex microimages combining phase-contrast and fluorescence. We used this tool to analyze the activity of the ER stress biosensor XBP1-TagRFP in response to tunicamycin in the 293A cell line, which is widely used in biomedical research. It was shown that this web-application allows you to quickly and with a low level of error process large arrays of microimages and obtain statistically reliable results. It is very important that this approach made it possible to obtain not only qualitative results, but also to detect quantitative changes in response to the ER stress inducer. The CellCountCV web-application is publicly available and can be used by a wide range of scientists who use genetically encoded biosensors based on fluorescent proteins in their research.

## Figures and Tables

**Table 1:** Validation Results Obtained on Images Selected from Two Series Never Seen by the Model.

Image Number	Predicted Cell Counts	True Cell Counts	Absolute Error (%)	Relative Error (%)
1	400	416	16	3.85
2	411	437	26	5.95
3	432	432	0	0.00
4	430	432	2	0.46
5	453	446	7	1.57
6	243	245	2	0.82
7	262	257	5	1.95
8	265	248	17	6.85
9	255	262	7	2.67
10	257	262	5	1.91
11	256	264	8	3.03
12	259	264	5	1.89
13	399	406	7	1.72
14	426	447	21	4.70
15	235	229	6	2.62
16	255	256	1	0.39
17	453	446	7	1.57
18	420	429	9	2.10
19	402	429	27	6.29
20	251	263	12	4.56
21	415	443	28	6.32
22	419	452	33	7.30
23	258	269	11	4.09
24	468	472	4	0.85
25	259	269	10	3.72
26	454	472	18	3.81
Total Counts:	9037	9247	Average relative error:	3.12

**Table 2:** Statistical Analysis of Red Cells Percentage Change at the End of ER stress Time Course ^1^.

Group 1	Group 2	U-Statistic	*P*-Value	Corrected *P*-Value
DMSO	Int	35.0	0.1365	0.8191
DMSO	TagRFP+	0.0	0.0001	0.0005
DMSO	Tu	3.0	0.0002	0.0013
Int	TagRFP+	0.0	0.0001	0.0005
Int	Tu	0.0	0.0001	0.0005
TagRFP+	Tu	8.0	0.0009	0.0051

^1^ The obtained differences in red cells percentages between the starting and the ending time points were compared with a non-parametric Mann–Whitney test (two-sided). Bonferroni *p*-value adjustment was used for multiple testing correction (N = 10).

**Table 3:** Results of Poisson GEE Regression with Autoregressive Covariance Structure ^1^.

Factor	Coefficient	SD	Z-Value	P > |Z|	[0.025]	[0.975]
Intercept	4.4028	0.050	87.781	0.000	4.305	4.501
DMSO	0.1446	0.080	1.813	0.070	−0.012	0.301
Tu	0.9292	0.043	21.452	0.000	0.844	1.014
TagRFP+	1.6325	0.035	46.401	0.000	1.564	1.701
Time	0.0083	0.001	14.710	0.000	0.007	0.009

^1^ 3880 observations, 40 clusters, cluster size = 97. Scale = 1.0. The model was built with the statsmodels package for Python. The basic negative control is the group of intact transfected ER stress-responsive cells (Int), DMSO—the group of transfected ER stress-responsive cells treated with DMSO; Tu—transfected ER stress-responsive cells treated with tunicamycin and TagRFP+—positive control—cell constantly producing TagRFP. Time—a factor of observation time.

**Table 4:** Statistical Analysis of Red Cells Percentage Changes at the End of the ER stress Time Course Between Different Control and Experimental Groups, Treated with Different DMSO and Tunicamycin Doses ^1^.

Group 1	Group 2	U-Statistic	*p*-Value	Corrected *p*-Value
DMSO_0.0	DMSO_0.5	132.0	0.1734	1.0
DMSO_0.0	DMSO_1.0	74.0	0.0012	0.0779
DMSO_0.0	DMSO_1.5	81.0	0.0032	0.2139
DMSO_0.0	Exp_Tu_0.0	116.0	0.0485	1.0
DMSO_0.0	Exp_Tu_10.0	0.0	0.0	0.0
DMSO_0.0	Exp_Tu_15.0	1.0	0.0	0.0
DMSO_0.0	Exp_Tu_5.0	76.0	0.0034	0.2212
DMSO_0.0	Pos_Tu_0.0	0.0	0.0	0.0
DMSO_0.0	Pos_Tu_10.0	0.0	0.0	0.0
DMSO_0.0	Pos_Tu_15.0	1.0	0.0	0.0
DMSO_0.0	Pos_Tu_5.0	0.0	0.0	0.0
DMSO_0.5	DMSO_1.0	83.0	0.0043	0.2857
DMSO_0.5	DMSO_1.5	90.0	0.0094	0.6222
DMSO_0.5	Exp_Tu_0.0	99.0	0.0149	0.9823
DMSO_0.5	Exp_Tu_10.0	0.0	0.0	0.0
DMSO_0.5	Exp_Tu_15.0	0.0	0.0	0.0
DMSO_0.5	Exp_Tu_5.0	71.0	0.0021	0.138
DMSO_0.5	Pos_Tu_0.0	0.0	0.0	0.0
DMSO_0.5	Pos_Tu_10.0	0.0	0.0	0.0
DMSO_0.5	Pos_Tu_15.0	0.0	0.0	0.0
DMSO_0.5	Pos_Tu_5.0	0.0	0.0	0.0
DMSO_1.0	DMSO_1.5	147.0	0.2806	1.0
DMSO_1.0	Exp_Tu_0.0	1.0	0.0	0.0
DMSO_1.0	Exp_Tu_10.0	0.0	0.0	0.0
DMSO_1.0	Exp_Tu_15.0	0.0	0.0	0.0
DMSO_1.0	Exp_Tu_5.0	0.0	0.0	0.0
DMSO_1.0	Pos_Tu_0.0	0.0	0.0	0.0
DMSO_1.0	Pos_Tu_10.0	0.0	0.0	0.0
DMSO_1.0	Pos_Tu_15.0	0.0	0.0	0.0
DMSO_1.0	Pos_Tu_5.0	0.0	0.0	0.0
DMSO_1.5	Exp_Tu_0.0	1.0	0.0	0.0
DMSO_1.5	Exp_Tu_10.0	0.0	0.0	0.0
DMSO_1.5	Exp_Tu_15.0	0.0	0.0	0.0
DMSO_1.5	Exp_Tu_5.0	1.0	0.0	0.0
DMSO_1.5	Pos_Tu_0.0	0.0	0.0	0.0
DMSO_1.5	Pos_Tu_10.0	0.0	0.0	0.0
DMSO_1.5	Pos_Tu_15.0	0.0	0.0	0.0
DMSO_1.5	Pos_Tu_5.0	0.0	0.0	0.0
Exp_Tu_0.0	Exp_Tu_10.0	1.0	0.0	0.0
Exp_Tu_0.0	Exp_Tu_15.0	3.0	0.0	0.0
Exp_Tu_0.0	Exp_Tu_5.0	105.0	0.0233	1.0
Exp_Tu_0.0	Pos_Tu_0.0	0.0	0.0	0.0
Exp_Tu_0.0	Pos_Tu_10.0	0.0	0.0	0.0
Exp_Tu_0.0	Pos_Tu_15.0	4.0	0.0	0.0
Exp_Tu_0.0	Pos_Tu_5.0	0.0	0.0	0.0
Exp_Tu_10.0	Exp_Tu_15.0	113.0	0.0625	1.0
Exp_Tu_10.0	Exp_Tu_5.0	114.0	0.0664	1.0
Exp_Tu_10.0	Pos_Tu_0.0	0.0	0.0	0.0
Exp_Tu_10.0	Pos_Tu_10.0	7.0	0.0	0.0
Exp_Tu_10.0	Pos_Tu_15.0	99.0	0.024	1.0
Exp_Tu_10.0	Pos_Tu_5.0	0.0	0.0	0.0
Exp_Tu_15.0	Exp_Tu_5.0	79.0	0.0045	0.2986
Exp_Tu_15.0	Pos_Tu_0.0	0.0	0.0	0.0
Exp_Tu_15.0	Pos_Tu_10.0	21.0	0.0	0.0003
Exp_Tu_15.0	Pos_Tu_15.0	126.0	0.1307	1.0
Exp_Tu_15.0	Pos_Tu_5.0	3.0	0.0	0.0
Exp_Tu_5.0	Pos_Tu_0.0	0.0	0.0	0.0
Exp_Tu_5.0	Pos_Tu_10.0	6.0	0.0	0.0
Exp_Tu_5.0	Pos_Tu_15.0	66.0	0.0013	0.083
Exp_Tu_5.0	Pos_Tu_5.0	0.0	0.0	0.0
Pos_Tu_0.0	Pos_Tu_10.0	53.0	0.0003	0.0197
Pos_Tu_0.0	Pos_Tu_15.0	2.0	0.0	0.0
Pos_Tu_0.0	Pos_Tu_5.0	51.0	0.0002	0.0156
Pos_Tu_10.0	Pos_Tu_15.0	45.0	0.0001	0.0075
Pos_Tu_10.0	Pos_Tu_5.0	149.0	0.3462	1.0
Pos_Tu_15.0	Pos_Tu_5.0	31.0	0.0	0.0012

^1^ The differences in red cell percentages between the starting and the ending time points were compared with a non-parametric Mann–Whitney test (two-sided). Bonferroni *p*-value adjustment was used for multiple testing correction. N = 18.

**Table 5:** Results of Poisson GEE Regression with Autoregressive Covariance Structure ^1^.

Factor	Coefficient	SD	Z-Value	*p* > |Z|	[0.025]	[0.975]
Intercept	3.9874	0.044	91.633	0.000	3.902	4.073
Tu	−0.0371	0.007	−5.026	0.000	−0.052	−0.023
Intron	−2.5533	0.128	−19.984	0.000	−2.804	−2.303
Tu:Intron	0.1336	0.012	11.556	0.000	0.111	0.156
Time	0.0816	0.001	54.663	0.000	0.079	0.085
Tu:Time	−0.0003	0.000	−1.368	0.171	−0.001	0.000
Intron:Time	0.0112	0.004	2.855	0.004	0.004	0.019
Tu:Intron:Time	−5.6 × 10^−5^	0.000	−0.138	0.890	−0.001	0.001

^1^ 3480 observations, 145 clusters, cluster size = 24. Scale = 1.0. DMSO-treated negative controls were excluded from the GEE analysis. The model was built with the statsmodels package for Python. The following factors were considered: Tu—tunicamycin concentration, Intron—intron present in the experimental group of cells and lacking in the positive controls, Time—a factor of observation time.

## Supplementary Materials

The following are available online at https://www.mdpi.com/1424-8220/20/13/3653/s1, Table S1: Contains the results of the first experiment with 3880 microscopic images (970 images per group with 10 fields of view per time point), Table S2: The results of the second experiment. 5208 microscopic images were processed.
